# Identification and characterization of the bacteriocin Carocin S3 from the multiple bacteriocin producing strain of *Pectobacterium carotovorum* subsp. *carotovorum*

**DOI:** 10.1186/s12866-020-01955-9

**Published:** 2020-09-01

**Authors:** Jyun-Wei Wang, Reymund C. Derilo, Ruchi Briam James S. Lagitnay, Huang-Pin Wu, Kai-In Chen, Duen-Yau Chuang

**Affiliations:** 1 Depertment of Gastroenterology, Chang Bing Show Chwan Memorial Hospital, 6 Lukon Road, Lukong Town, Changhua, 505 Taiwan; 2 Department of Chemistry, National Chung-Hsing University, 250, Kuokuang Rd, Taichung City, 402 Taiwan; 3 Division of Pulmonary, Critical Care and Sleep Medicine, Chang Gung Memorial Hospital, Keelung, Taiwan; 4 College of Medicine, Chang Gung University, Taoyuan, Taiwan

**Keywords:** *Pectobacterium carotovorum* subsp. *carotovorum*, Low-molecular-weight Bacteriocin, Carocin S3

## Abstract

**Background:**

*Pectobacterium carotovorum* subsp. *carotovorum* belongs to the *Enterobacteriaceae* family, which causes soft-rot disease in numerous plants worldwide resulting in significant economic losses. Results from our previous studies showed that the strain H-rif-8-6 produces low-molecular-weight bacteriocin (LMWB) Carocin S1. Interestingly, TH22–10, the *caroS1K:Tn5* insertional mutant in H-rif-8-6, loses Carocin S1 producing ability, but still produces other LMWBs which the indicator strain SP33 can detect. The SP33 is one of the many strains that are sensitive toward the cytotoxic effects of Carocin S3K, but not Carocin S1. The result revealed that H-rif-8-6 is a multiple-bacteriocin producing strain.

**Results:**

In this study, a 4.1-kb DNA fragment was isolated from the chromosomal DNA of Pcc strain, H-rif-8-6, by a DNA probe using the *caroS1K* gene as the template. DNA sequencing and analysis by GenBank revealed two complete open reading frames (ORFs), designated ORF1 and ORF2, which were identified within the sequence fragment. ORF1 and ORF2, similar to the identified *carocin S2* genes, encode the killer (Carocin S3K) and the immunity (Carocin S3I) proteins, respectively, which were homologous to the *colicin E3* gene. Carocin S3K and Carocin S3I were expressed, isolated, and purified in *Escherichia coli* BL21 after subcloning of the expression plasmid pGS3KI or pGSK3I. SDS-PAGE analysis showed that the relative masses of Carocin S3K and Carocin S3I were 95.6 kDa and 10.2 kDa, respectively. The results reveal that Carocin S3K has higher antimicrobial and specific antimicrobial activities for Pcc along with a nuclease activity than Carocin S3I. However, Carocin S3I inhibits the activity of Carocin S3K. Interestingly, a high concentration of Carocin S3I protein is also a DNA nuclease, and Carocin S3K also inhibits its activity.

**Conclusion:**

This study showed that another type of bacteriocin was found in *Pectobacterium carotovorum.* This new type of bacteriocin, Carocin S3, has the killer protein, Carocin S3K, and the immunity protein, Carocin S3I.

## Background

*Pectobacterium carotovorum* subsp. *carotovorum* (Pcc), also called *Erwinia carotovora* subsp. *carotovora*, is a member of the soft rot *Enterobacteriaceae* (SRE) family and causes soft-rot disease resulting in economic losses in a wide variety of plants worldwide. The *Pectobacterium carotovorum* subspecies are characterised by their ability to produce high levels of extracellular enzymes such as pectate lysase (Pel), polygalacturonase (Peh), cellulase (Cel), and protease (Prt). The major pathogenicity determinants are an arsenal of extracellular pectinases, including several pectate lyase isozymes, pectin lyase, pectin methylesterase, and pectin polygalacturonase, which degrade plant cell wall components, thereby leading to tissue maceration and cell death [1, 2]. Furthermore, a range of other degradative enzymes such as cellulase and protease are secreted, but their role in causing virulence is uncertain [3].

Bacteriocins are bactericidal and extracellular toxins produced by both gram-positive and gram-negative bacteria [4–6]. These bacteriocins destroy closely related bacteria, but not the producer strain itself. According to Klaenhammer, 99% of all bacteria produce at least one bacteriocin [7, 8]. All major groups of bacteria produce these inhibitors [3], and the susceptible cell is recognised by specific target receptors present on the cell membrane. The colicin family produced by *Escherichia coli* is divided into DNase (Colicins E2, E7, E8 and E9), RNase (Colicins E3, E4 and E6), and pore-forming Colicins (Colicins A, E1, Ia and Ib) [4, 9]. Some Pcc species produce one or more antibacterial substances, also containing bacteriocin, which enhances their competency against other related bacterial species. According to Kikumoto et al., Nakatani et al., and Tsuyama et al., the antibacterial activities of two types of bacteriocins produced by avirulent bacteriocin-producing biocontrol agents may contribute to the suppression of soft-rot disease [10–12].

Some Pcc species produce high-molecular-weight bacteriocins (HMWBs or large bacteriocins), such as the Carotovoricin Er, which have similar structures with those of bacteriophages [3, 11, 13, 14]. The HMWB Carotovoricin Er has a bulky antenna-like tail, an inner core and a cylindrical contractile structure, and displays an inhibitory activity. Comparatively, low-molecular-weight bacteriocins (LMWBs or small bacteriocins) such as Carocin are small polypeptides similar to Colicin. Carocin S1 and Carocin D are deoxyribonuclease types of LMWBs, and Carocin S2 is a ribonuclease type of LMWB [3, 6, 14]. Moreover, Carocin S1 is secreted extracellularly through the type III secretion system, which also controls the cell motility of the bacterium [15].

O’Shea et al. suggested that some gastrointestinal strains of *Lactobacillus salivarius* may produce multiple bacteriocins from a single locus [16], but no evidence was provided to support the hypothesis. However, almost all bacteriocin-producing gram-negative bacteria strains produce one bacteriocin protein, and the encoded gene is located at the plasmid.

From our previous studies of Carocins, we found that the Carocin S1-producing strain, H-rif-8-6, does not only produce one LMWB but is also capable of secreting multiple bacteriocins. The *caroS1K:Tn5* strain TH22–10 has shown its antibiotic ability using the indicator strain SP33, the same strain used as an indicator for Carocin S2, but not for Carocin S1 (Fig. 2) [3]. However, it has not shown activity against Ea1068. This antibiotic activity from TH22–10 was also confirmed by trypsin-treatment experiment (data not shown). The results showed that the antibiotic activity was not from a bacteriophage but could be from an LMWB. Moreover, the western blotting experiment for anti-CaroS1K revealed that the activity was not from the Carocin S1K. Hence, these results indicated that the H-rif-8-6 strain is a possible multi-bacteriocin producer.

**Fig. 1:**
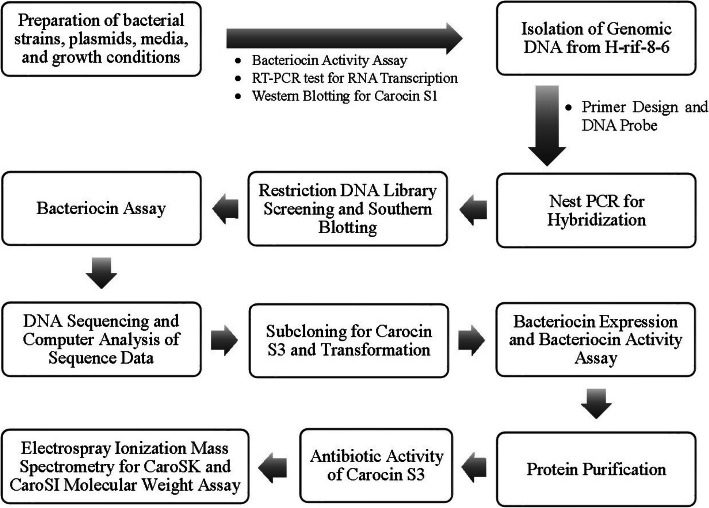
Flow diagram of the research process. To confirm the presence of another bacteriocin from Pcc, we initially subjected the TH22–10 (*caroS1K:Tn5*) to bacteriocin activity assay, RT-PCR, and western blotting. After confirming that the Pcc strain H-rif-8-6 produces another bacteriocin apart from the previously identified bacteriocin, Carocin S1, the genomic DNA from H-rif-8-6 was isolated and digested using several restriction enzymes and was assayed with the best restriction enzyme for creating DNA libraries as demonstrated by the Southern blotting experiment. Then, bacteriocin production was tested through the bacteriocin activity assay. After that, the DNA nucleotide sequence and the deduced amino acid sequence of other known bacteriocins were compared. Subsequently, subcloning for the novel bacteriocin, designated as Carocin S3, and transformation were performed. Recombinants were used to express the Carocin S3 proteins, Carocin S3K and Carocin S3I. Furthermore, bacteriocin expression and bacteriocin activity assays were performed. Also, protein purification and Carocin S3 antibiotic activity test were carried out. Finally, Electrospray Ionization Mass Spectrometry Molecular Weight Assay was done to determine the molecular weights of the killer protein, Carocin S3K*,* and immunity protein, Carocin S3I

This present study aimed to investigate the presence of another LMWB from the H-rif-8-6 strain, which was hypothesised to be a multi-bacteriocin producer. Moreover, this paper aimed to prove that the strain is capable of secreting other bacteriocins aside from the previously discovered Carocin S1. In this paper, we report the cloning and sequencing of DNA encoding one LMWB in Pcc designated “Carocin S3”, which was introduced into an expression plasmid encoding two proteins, Carocin S3K and Carocin S3I. These proteins were purified and characterised, and their primary activities of killing (Carocin S3K) and immunity (Carocin S3I) were studied in vivo and in vitro. A flow diagram of the research process is summarised in Fig. 1.

## Results

### H-rif-8-6 is a multi-LMWB producer

**Fig. 2:**
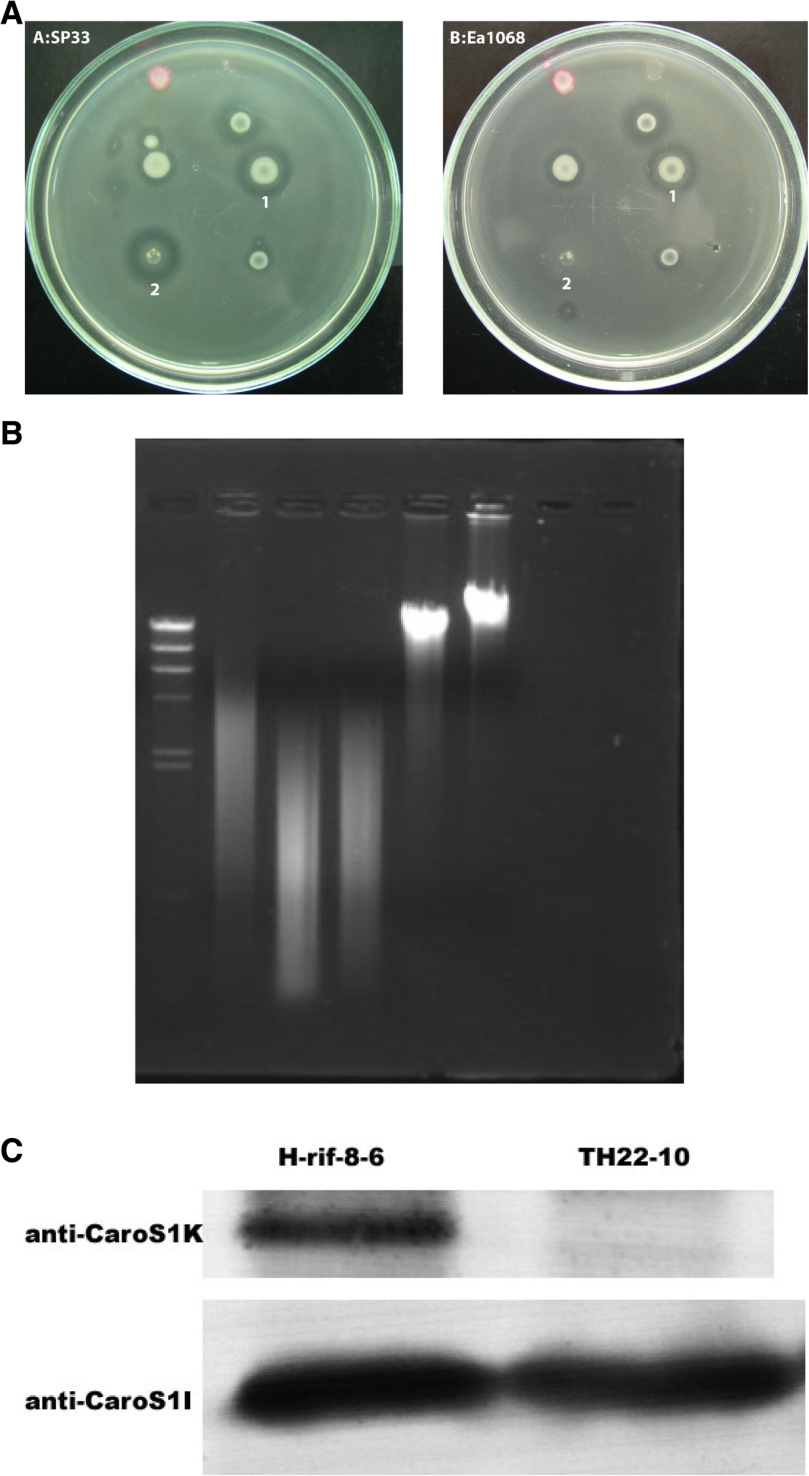
**A** Bacteriocin assays of *Pectobacterium carotovorum* subsp. *carotovorum*. Numbered strains show the wild type H-rif-8-6 (1) and the *caroS1K:Tn5* insertion mutant, TH22–10 (2). Other unlabeled strains are Tn5 insertion mutants of progeny of H-rif-8-6 strain. The indicators were Pcc strain SP33 (A) and Ea1068 (B). **B** In vitro analysis of the DNase activity of rough protein extract from TH22–10. Samples were subjected to gel electrophoresis and labeled as follows: Lane M, the *Hind*III-digested λ DNA marker; 1, the equal quantities of *EcoR*I-digested genomic DNA; 2, the genomic DNA with protein extract from H-rif-8-6; 3, the genomic DNA with protein extract from TH22–10; 4, the genomic DNA only with reaction buffer; 5, the genomic DNA only. **C** Carocin S1 protein detection. Carocin S1K antiserum antibody (anti-CaroS1K) and Carocin S1I antiserum antibody (anti-CaroS1I) were detected from rabbits

To confirm that H-rif-8-6 is a multi-LMWB producer, we assayed the bacteriocin activity from the *caroS1K:Tn5* strain TH22–10. The results showed that TH22–10 could inhibit SP33 growth but not Ea1068 (Fig. 2A). Further isolation of rough protein from TH22–10 and testing its function indicated that the cell extract contains nuclease (Fig. 2B), and western blotting analysis showed that this nuclease is not Carocin S1K (Fig. 2C).

### Homologous analysis of Carocin S3 with others’ LMWB nuclease activity

Amino acid sequence analysis of Carocin S3, Pyocin S3, and Pyocin AP41 was observed (see Supplementary Fig. 1). Highly homologous DNA fragments, i.e., LMWB obtained from H-rif-8-6, exhibited DNase activity based on amplified nested-PCR product. The amino acid sequence homology analyses of Carocin S3, Pyocin S3, and Colicin E3 were performed using DNASIS MAX 3.0 software. The results showed highly homologous sequences from the 360th to 568th amino acids.

We designed 2 primers (listed in Table 2), CaroS1_for2 and CaroS1_rev2, to amplify the DNA fragment by PCR experiment and subcloned this fragment into the pGEM T-easy vector for preparing the DNA probe CAROS.

### DNA libraries demonstrator and LMWB gene isolation

Chromosomal DNA from the H-rif-8-6 strain was isolated and digested using several restriction enzymes. Furthermore, it was assayed with the best restriction enzyme for creating DNA libraries, as demonstrated by the Southern blotting experiment. The results showed that an approximately 4.5-kb DNA fragment was detected after *Bgl*II digestion, which was isolated from agarose gel and subcloned into pBR322 vector for demonstrating DNA libraries. Further, over 350 colonies were isolated from the DNA libraries assayed for bacteriocin activities followed by the sequencing of their DNA. As a result, 11 colonies displayed LMWB activity, and only one was possibly a new LMWB gene after DNA sequencing. This vector was named pBRS3, and the corresponding gene was called *carocin S3*.

DNA sequencing of the pBRS3 vector revealed the complete 4143-bp DNA sequence. Moreover, further analysis of the 4143-bp gene *carocin S3*, indicated 2 ORFs. These are the 2640-bp long gene, *caroS3K*, and a smaller 270-bp gene, *caroS3I*. The stop codon (TGA) of *caroS3K* overlaps the start codon of *caroS3I* by 4-bp (ATGA). Presumably, annotation of the amino acid sequences was deduced from the *carocin S3* gene by DNASIS MAX 3.0 software, which was comparable to other analogous proteins searched using the BLAST and FASTA programs.

It could be deduced from the nucleotide sequence that ORF1 codes for a protein of 880 amino acids, which shows higher homology with Carocin DK (73%), and Carocin S2K (78%), the killer protein of *Pectobacterium carotovorum*. The ORF2 encodes 90 amino acids and shows homology with the possible immunity protein of LMWB in the *Yersinia pestis* PY-64 strain (GenBank accession number: EIS53281). It was revealed that *caroS3K* is an antibiotic-producing gene, which codes for a protein containing 880 amino acids showing a deduced molecular mass of 95.6 kDa. Similarly, *caroS3I*, a product of the *caroS3K*-immune gene, contains 90 amino acids, displaying a deduced molecular mass of 10.2 kDa. Notably, higher homologies between *caroS3K* and the other genes were found at the C-terminal end, which was thought to be the catalysis centre of ribonuclease. FASTA program revealed that the slitting of a segment at Gly507 toward the end of Carocin S3K displayed close to 80% similarity with Carocin D and 91% similarity with Carocin S2K. Interestingly, Carocin DK belongs to the DNase group of bacteriocins, whereas Carocin S2 belongs to the RNase group.

### Purification and characterisation of Carocin S3

Purified protein obtained from the pEN3KI construct, which contained *caroS3KI* encoded into the pET32a vector, was devoid of killer activity. On deleting a series of tag attachments ahead of the *caroS3K* sequence, which made the plasmid pEN3KI merely conserve the T7 promoter, the purified protein expressed activity. With the (His)6-tag flag, the *caroS3I*-expressed plasmid pES3I was constructed using the same method.

**Fig. 3:**
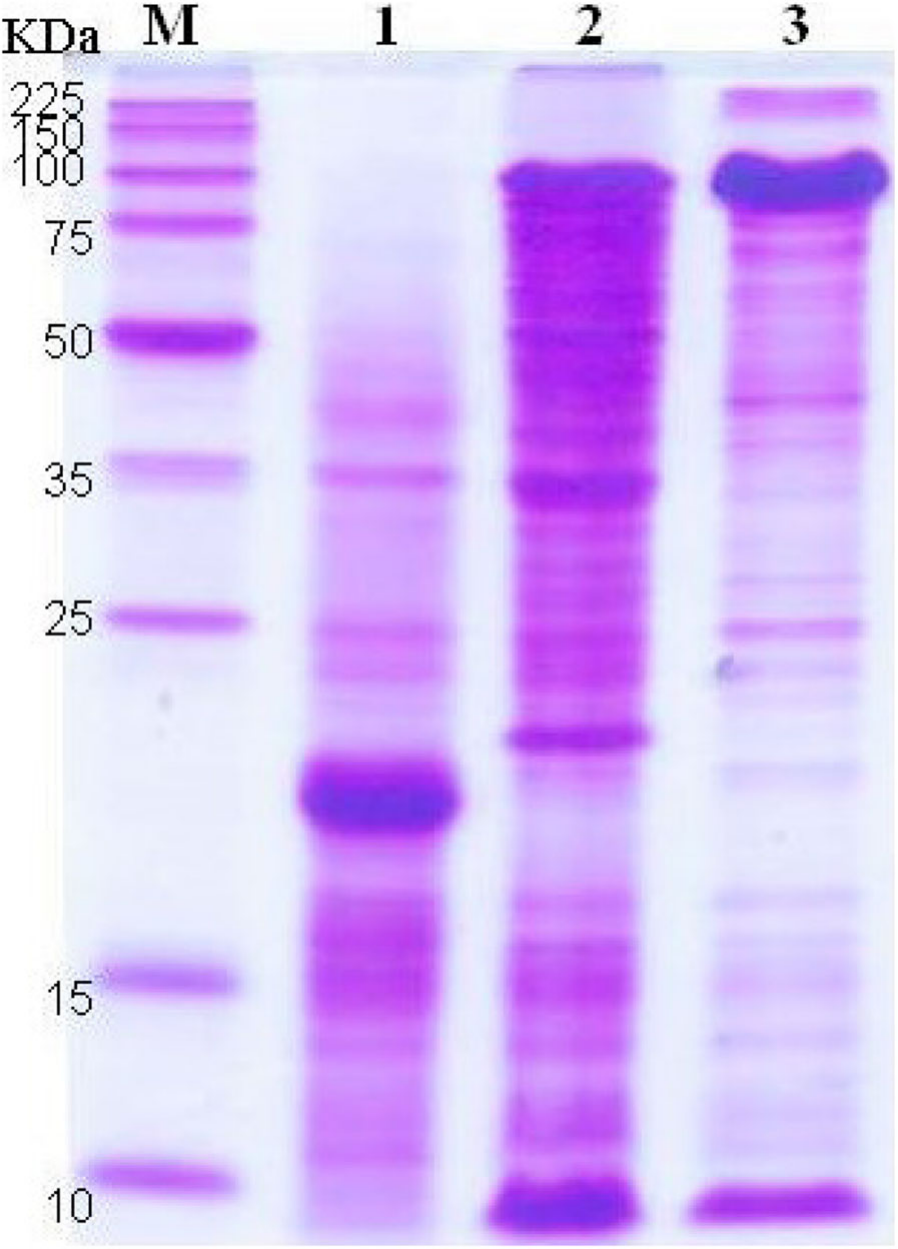
Carocin S3 protein analysis by 15% SDS-PAGE and comassie-blue stained. M, protein molecular weight marker; 1, BL31/pET32a (control); 2, BL21/pES3KI cells; 3, BL21/pES3KI protein extracts. The molecular weight of Carocin S3K is 95.6 kDa, and Carocin S3I is 10.2 kDa

*E. coli* BL21 (DE3) recombinants, transformed with either pEN3KI or pES3I, were used to express the Carocin S3 proteins, Carocin S3K and Carocin S3I. All transformants were induced with IPTG under the control of the T7 promoter. The proteins were isolated and quantified by 40 ~ 50% ammonium sulfate precipitation followed by chromatographic separation of cell lysate. SDS-PAGE gels of purified Carocin S3 stained with Coomassie blue (described in Fig. 3) showed a lane containing the protein marker (Lane M) whose sizes (in kiloDalton) are indicated on the left. Elution of the column with a salt-gradient led to an expected band of Carocin S3K at a Mr. of ~ 95.6 kDa on the gel. Figure 3 displays the purification of Carocin S3K with the arrowheads pointed toward enriched fractions. Carocin S3I was purified by using the same procedure. A relatively stronger residual band is indicated by another arrowhead, which was predicted to have a Mr. of 10.2 kDa.

**Fig. 4:**
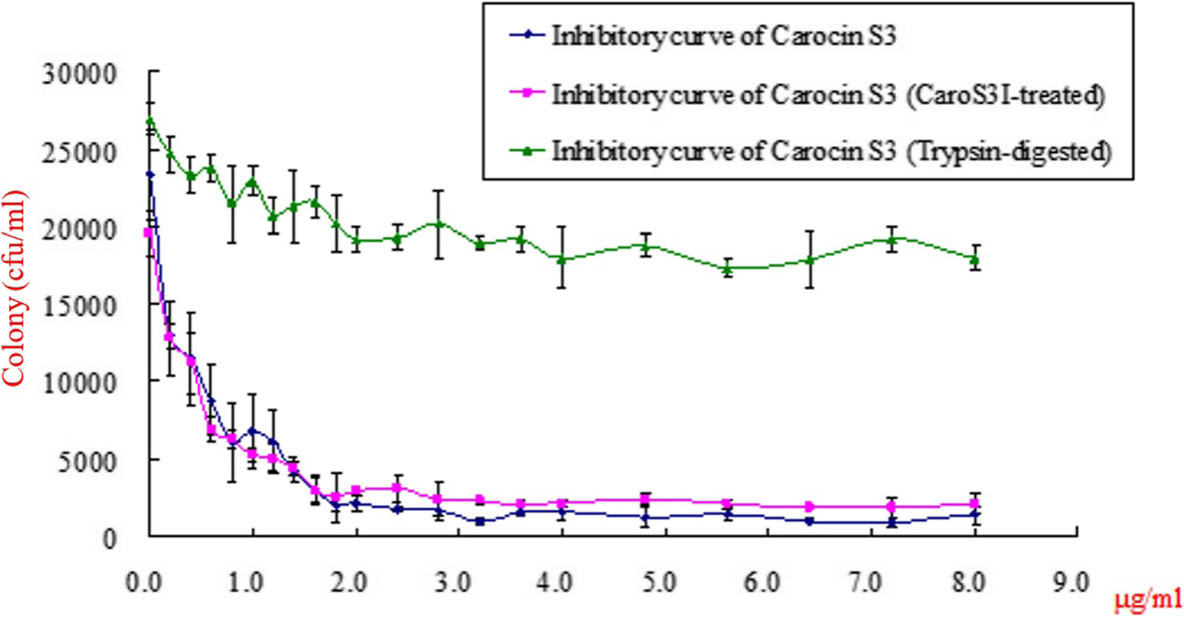
Survival of SP33 cells treated with Carocin S3. Aliquots of indicator SP33 cells were treated with increasing concentrations of Carocin S3K ( ) and Carocin S3K:Carocin S3I in a molar ratio of 1:1 ( ). The effect of trypsin on the Carocin S3K was also assayed ( ). The data are reported as means ± standard deviations

The purified Carocin S3K involved in the growth inhibition of susceptible indicator SP33 was characterised. The cell survival experiment was carried out by pre-incubating Pcc strain SP33 with Carocin S3K at 28 °C for 60 min. After inoculating onto LB agar medium, the surviving cells were counted. The survival number decreased at increasing concentrations of Carocin S3K (Fig. 4). This effect intensified with adding an initial concentration of 2.0 μg/mL, and the surviving number of cells drastically decreased. Cell populations did not survive at a final concentration of 4 μg/mL.

### ESI-MS spectra for Carocin S3I from purified Carocin S3

**Fig. 5:**
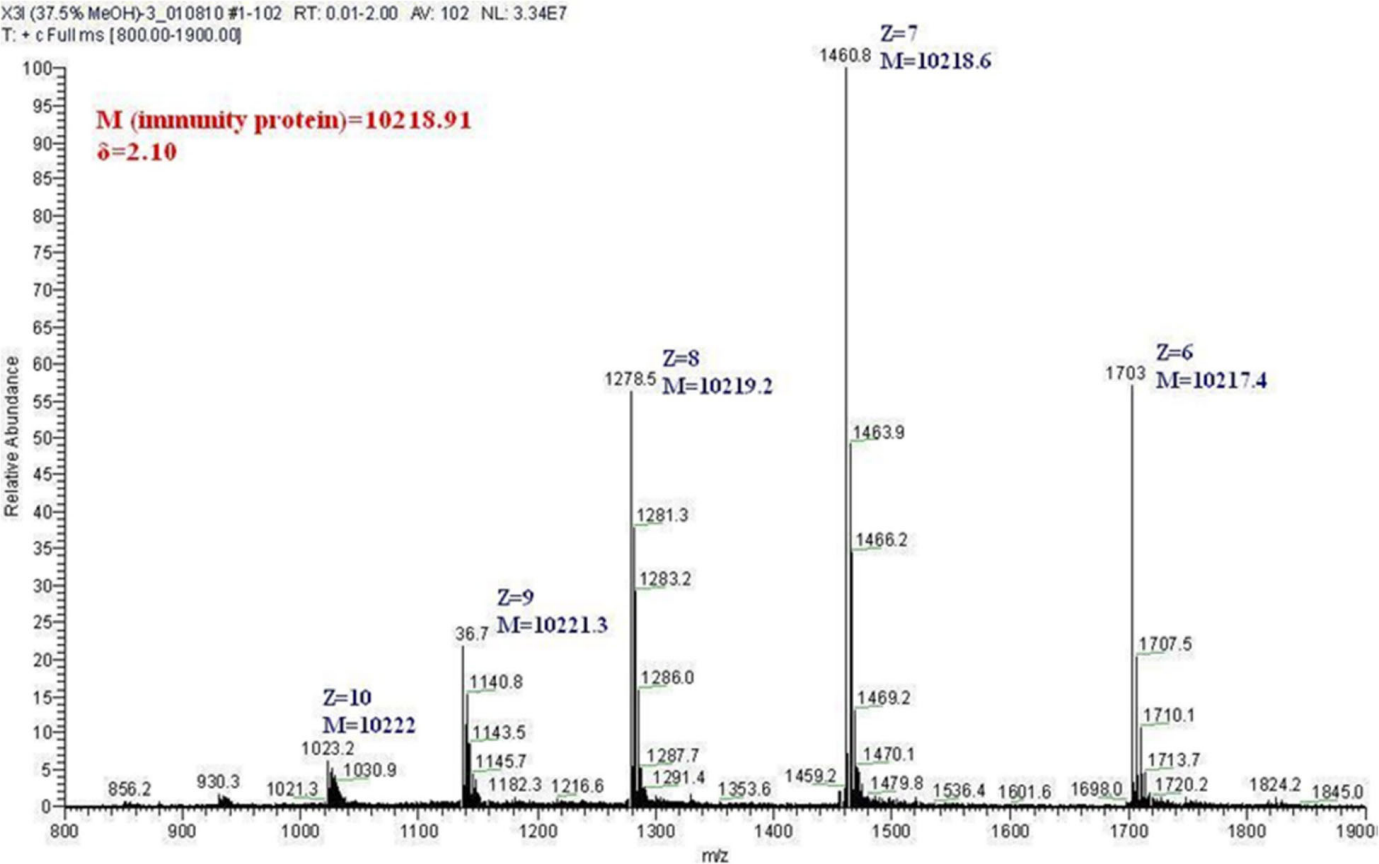
Molecular weight analysis for Carocin S3I from Carocin S3 by ESI-MS

The migration of Carocin S3I from Carocin S3 on SDS/PAGE corresponded to a protein of MW 10000–11,000, which is almost 1000 units greater than the anticipated MW deduced from the amino acid sequence (MW = 10.2 kDa). To determine whether this high value was a result of posttranslational modification or simply the migration property of Carocin S3I on SDS gel, we determined the monomeric molecular mass of Carocin S3I by ESI-MS. This technique is now widely used to determine the MW of proteins. The observed MW of isolated CaroS3I is 10,218.91 ± 2.10 (Fig. 5), as determined from two independent experiments. It should be emphasised that the immunity protein is undergoing posttranslational modification after binding to the killer protein.

**Fig. 6:**
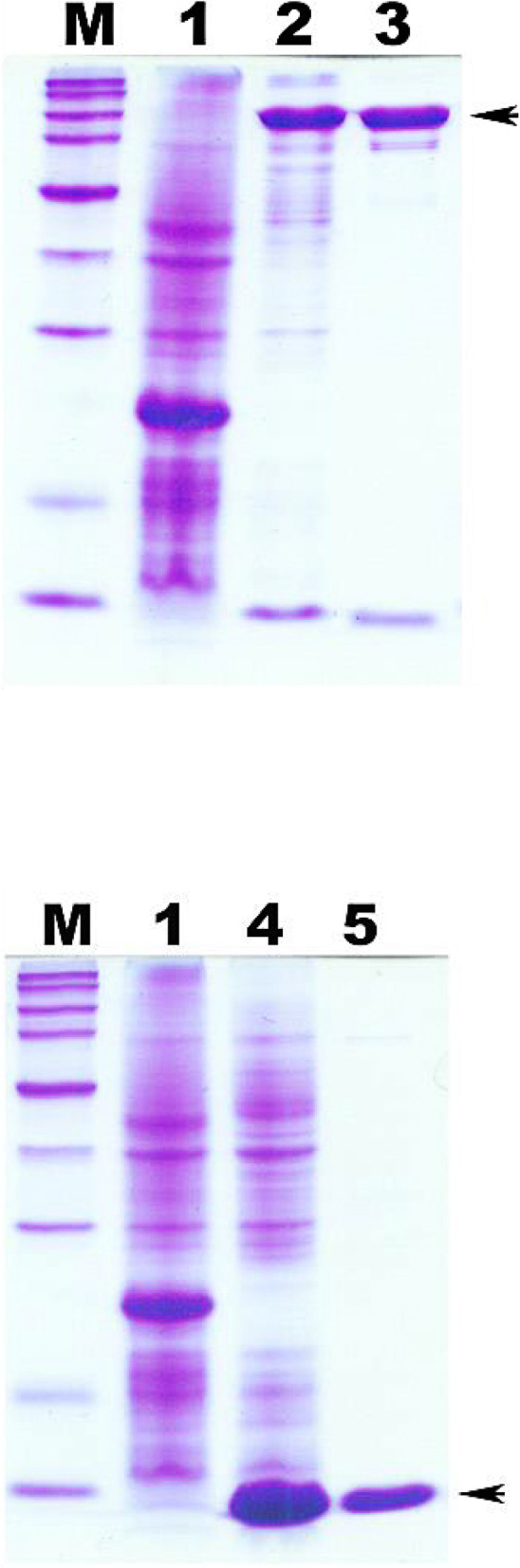
SDS-PAGE analysis of purified protein. Shown are the Carocin S3K (**a**) and Carocin S3I (**b**). Samples were subjected to electrophoresis in 10% polyacrylamide gels, which were stained with Coomassie blue. These were labeled as follows: lane M, molecular weight standards (kDa); lane 1, cell lysate of *E. coli* BL21/pET32a; lanes 2 and 4, IPTG-induced cell lysates of BL21/pES3kI and BL21/pES3I, respectively; lanes 3 and 5, a purified protein obtained after elution. The arrowheads indicate the killing protein of Carocin S3K (**a**) and the immunity protein of Carocin S3I (**b**)

The immunity protein probably interacts with the killer Carocin S3K. Loading the purified Carocin S3 complex, we observed a band that co-electrophoresed with the immunity protein on SDS acrylamide gel (Fig. 6). This result suggests that the two proteins actively interact with each other, which demonstrated that, for Carocin S3K, the bound immunity protein is not a prerequisite for cell attachment or translocation, because the free Carocin has precisely the same bactericidal activity as the Carocin complexed to its immunity protein.

### Nuclease activity of Carocin S3

The homology comparison of Carocin S3 showed that Carocin S3K was a certain kind of nuclease. Thus, the genomic DNA from the indicator strain Ea1068 was extracted after incubation with the purified protein Carocin S3K. Electrophoretic patterns showed that Carocin S3K significantly digested deoxyribonucleic acids, similar to the digestion of *EcoR*I-digested genomic DNA (Fig. 6a). According to Bradley’s classification, Carocin S3 is also an LMWB [2]. The substrate and gene structure of Carocin S3 were similar to those of Carocin D [14] and Carocin S2 [6] produced by Pcc species. The two genes, *caroS3K* and *caroS3I*, code for the 95.6-kDa and 10.2-kDa components of Carocin S3, respectively.

Furthermore, to examine the killer activity of Carocin S3K, it was mixed with an equal mass of Carocin S3I, whose immune activity could be observed in vitro. The quantity of digested segments (lane 6), shown in lanes 3 and 4, dramatically disappeared. Thus, it could be verified that Carocin S3I significantly inhibited the killer activity of Carocin S3K.

**Fig. 7:**
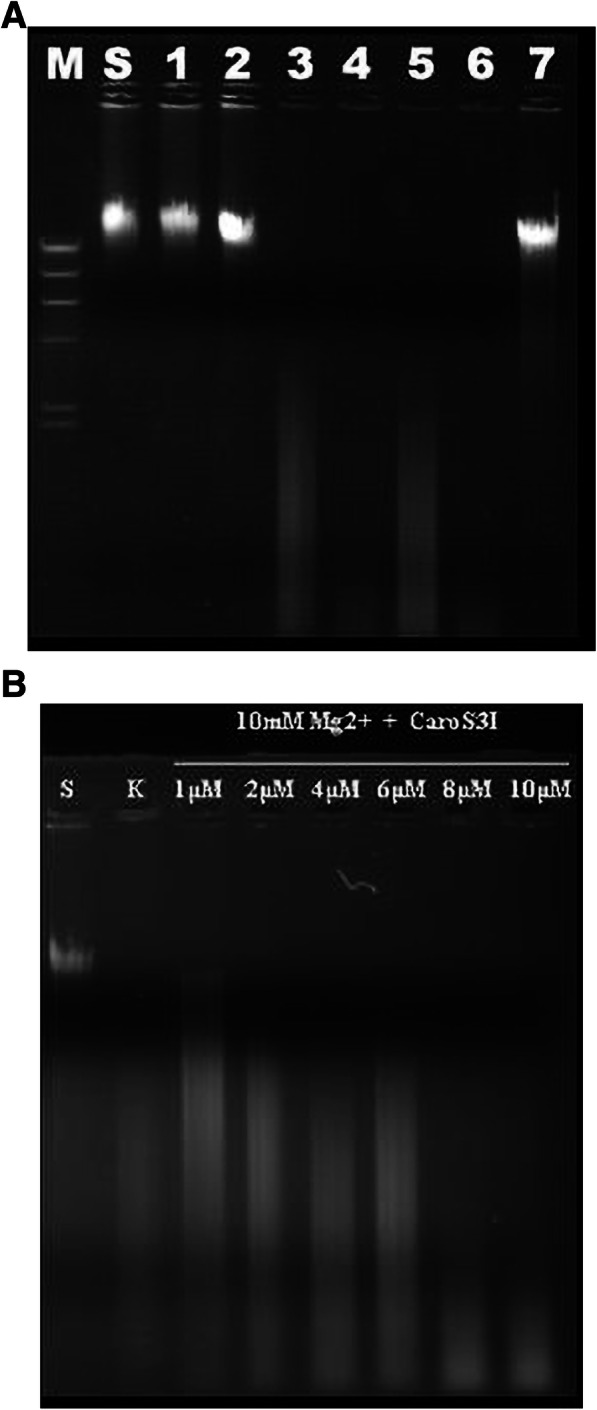
In vitro hydrolysis of DNA and RNA by Carocin S3. **a** Analysis of the DNase activity of Carocin S3. The samples are labelled as M, marker; S, genomic DNA of Ea1068; 1, genomic DNA/ddw; 2, genomic DNA/buffer (contains Mg^2+^); 3, genomic DNA/*BamH*I + buffer; 4, genomic DNA/*EcoR*I + buffer; 5, genomic DNA/Carocin S3I (1 μM) + buffer; 6, genomic DNA/Carocin S3K(1 μM) + buffer; 7, genomic DNA/Carocin S3K(1 μM) + Carocin S3I(1 μM) + buffer. **b** 0.15 μg of genomic DNA per sample were incubated with different concentration of Carocin S3I. Lane S, contains 0.15 μM genomic DNA; lane K, 0.15 μM genomic DNA and 1 μM Carocin S3K; all other lanes contain 0.15 μM genomic DNA and different concentrations of Carocin S3I protein. All samples contained 10 mM Mg^2+^

Interestingly, Carocin S3I not only inhibited Carocin S3K activity, but Carocin S3I alone could also digest deoxyribonucleic acid (Fig. 7a and b). However, the nuclease efficiency of Carocin S3I was considerably lower than that of Carocin S3K.

### Nucleotide sequence accession number

The Genbank accession number of the sequence of the *carocin S3* gene is KC848768.

## Discussion

In this study, we successfully isolated a new LMWB *carocin S3* gene, which is borne by the chromosomal gene encoding bacteriocin. We also showed that the Pcc H-rif-8-6 is a multi-LMWB producer, which produces Carocin S1 and Carocin S3 proteins. O’Shea et al. suggested that some gastrointestinal strains of *Lactobacillus salivarus* produce multiple bacteriocins from a single locus that are localised to mega-plasmids [16]; however, no evidence has been provided so far, and our study may be the first to confirm this hypothesis.

Based on the sequence analysis, Carocin S3 comprises 2 overlapping ORFs, *caroS3K* and *caroS3I*. A putative Shine-Dalgarno sequence 5ˊ-AUGGA-3ˊ, which has also been observed in the DNA sequence of *carocin S1* and *carocin S2*, is located upstream (− 9 bp to − 13 bp) of the start codon AUG, suggesting that it could be a ribosome binding site for *caroS3K* [3, 6]. Comparison of the upstream sequences of both *caroS3K* and *caroS3I* has shown that the 2 consensus sequences, 5ˊ-TATAAAAA-3ˊ (− 34 bp to − 41 bp) and 5ˊ-GAAGT-3ˊ(− 61 bp to − 65 bp), are both located upstream of the start codon. These results are also very similar to the *carocin S2* gene. Presumably, 5ˊ-TATAAAAA-3ˊ is the − 10 promoter and 5ˊ -GAAGT-3ˊ is the − 35 promoter for the *carocin S2* gene, although they differ from those of *E. coli* [17, 18].

This study investigates the basis for biochemical immunity toward the DNase *caroS3K* obtained from the Pcc species. In this article, we have described the purification and properties of the unbound killer protein and the protein responsible for providing immunity to Carocin S3K. According to Bradley’s classification, Carocin S3 is also an LMWB [3]. The substrate and gene structure of Carocin S3 were similar to those of Carocin D [14] and Carocin S2 [6] produced by Pcc species. The 2 genes, *caroS3K* and *caroS3I*, code for the 95.6-kDa and 10.2-kDa components of Carocin S3, respectively.

The in vivo activity revealed that in the presence of Carocin S3I, Carocin S3K does not demonstrate any cytotoxic effect against Pcc SP33, a strain that is usually sensitive toward the cytotoxic effects of Carocin S3K. Irrespective of its mechanism of action, the immunity protein cannot pass into Carocin-sensitive cells or even prevent the attachment of Carocin to its cell surface receptors. If these were possible, the treatment of Carocin S3I with Carocin S3K in vitro would result in an active protein in vivo. Such changes are expected for a molecule whose role is to protect Carocin-producing cells, but not cells that are targeted by Carocin. Results of the ESI-MS assay and Carocin S3K inhibition activity assay reveal that Carocin S3I recruited Carocin S3K to form a new complex in which Carocin S3K loses its DNA nuclease activity. In contrast, Carocin S3I alone worked also as a DNA nuclease, and its activity was inhibited when combined with Carocin S3K. The recruit mechanism between Carocin S3K and Carocin S3I is interesting and needs further investigation.

**Fig. 8:**
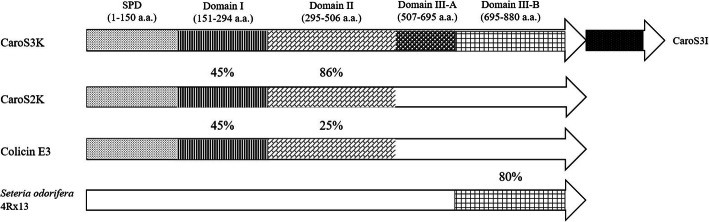
Region similarity of the putative domains of the Carocin S3 with those of related bacteriocins. The related ORFs are shown. Percentage values indicate the percent relatedness to the corresponding regions in Carocin S3. The length of each domain is proportional to the number of amino acids, and homologous domains are shaded similarly

The amino acid sequence of Carocin S3K has a signal peptide region (SPD) and 3 putative domains (Fig. 8). The SPD (1 ~ 150 a.a.) does not share sequence similarity with any other protein, as indicated by the FASTA homologous search performed from the Swiss-Port. It is regarded as the signal peptide domain, which may act as a signal for extracellular secretion by type III secretion system and is highly homologous in the same region of Carocin S2. Interestingly, on attempts to delete SPD of Carocin S3K, we found that Carocin S3K cannot be transported outside the cell like the FliC-KO strain [13].

Domain I (151 ~ 294 a.a.) is regarded as the translocation domain and is approximately 45% homologous to the translocation domains of Carocin S2K and Colicin E3 (data not shown). Presumably, it directs the cytotoxic domain to the periplasmic space [18, 19], suggesting that this domain is likely Domain I of Carocin S2K and also carries the putative TonB box (a sequence recognition motif DTMTV) found in the N-terminal domain of Carocin S3K, which is thought to participate in bacteriocin translocation [9]. Thus, we suggest that Carocin S3 could also be a TonB-dependent bacteriocin.

Domain II (295 ~ 506 a.a.) is implicated as the receptor-binding domain and was found to be approximately 83% homologous to the receptor-binding domain of Carocin S2 and 25% to Colicin E3.

With regards to the C-terminal amino acid sequence from 507 ~ 880 a.a. of Carocin S3, two sub-regions were separated, Domain III-A (507 ~ 695 a.a.) and Domain III-B (695 ~ 880 a.a.). However, the function of Domain III-A is still unknown and needs further investigation. Domain III-B showed high sequence homology (80%) to the H-N-H endonuclease domain protein of *Serratia odorifera* 4Rx13, and the H-N-H motif was easily found in some endonucleases belonging to different species.

However, the amino acid sequence of Carocin S1 was found different from the sequences of Carocin D, Carocin S2, and Carocin S3. Both Carocin S1 and Carocin S3 are produced from the same strain, H-rif-8-6, which suggests that Carocin S1 is possibly a different type of LMWB.

The amino acid sequences of *caroS31* structural genes were homogenously aligned using BLAST, and the results did not show any related structural functions (Supplemental Fig. 1). However, related literatures about bacteriocins, such as Pyocin and Colicin, suggest that immunity protein comes after the killer protein. Hence, we reasonably speculate that this part is the Carocin S3 immunity gene, *caroS31*.

**Fig. 9:**
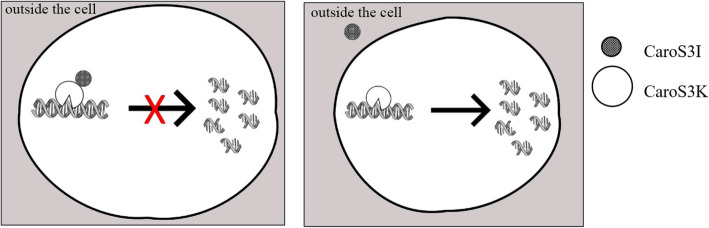
The DNA nuclease activity of *CaroS3I* and *CaroS3K* in the producer cell (**a**) and target cell (**b**)

In Fig. 9, the producer cell contains Carocin S3K and Carocin S3I. The Carocin S3K/Carocin S3I complex did not exhibit DNA nuclease activity inside the producer cell (Fig. 9a). The in vitro assay demonstrated that the producer cell releases Carocin S3K to the target cell, which exhibited nuclease activity against the target cell’s DNA. Similarly, when the producer cell releases Carocin S3I to the target cell, it also caused damage to the target cell’s DNA but not as severe as the Carocin S3K. To further investigate the nuclease activity, in vitro assay of the mixed Carocin S3K and Carocin S3I was done. Both Carocin S3K and Carocin S3I left the producer cell, but only Carocin S3K entered the target cell leaving the Carocin S3I outside (Fig. 9b). The result showed that Carocin S3I loses its nuclease activity.

Both Carocin S3I and Carocin S3K have DNA nuclease activity. However, they lose their nuclease activities when mixed. The protein function of Carocin S3I needs to be further investigated.

## Conclusion

A new LMWB *carocin S3* gene, which is borne by the chromosomal gene encoding bacteriocin, was successfully isolated. This study proves that the Pcc H-rif-8-6 is a multi-LMWB producer, producing two bacteriocins, Carocin S1 and Carocin S3.

Based on the biochemical immunity, it was found out that Carocin S3K has immunity toward DNA nuclease. Carocin S3K demonstrated that the bound immunity protein is not a prerequisite for cell attachment or translocation due to the free Carocin, which has similar bactericidal activity as the Carocin complex to its immunity protein. Pcc strain SP33 usually is sensitive to the cytotoxic effects of Carocin S3K. However, with the presence of Carocin S3I, Carocin S3K loses its cytotoxic activity. Regardless of the mechanism of action, the immunity protein, Carocin S3I*,* can neither pass through the Carocin-sensitive cells nor inhibit the attachment of Carocin to the cell surface receptors.

Homologous alignment of the amino acid sequence of Carocin S3I using BLAST did not show any related functions. However, it can be reasonably deduced from related studies on other bacteriocins that the gene corresponds to the *carocin S3* immunity gene.

Lastly, the amino acid sequence of Carocin S3 is unique when compared against the sequences of Carocin D, Carocin S1, and Carocin S2. Both Carocin S1 and Carocin S3 are produced from the same strain, H-rif-8-6, which further suggests that Carocin S3 is a different type of LMWB.

## Methods

### Bacterial strains, plasmids, media, and growth conditions

The strains and plasmids used in the present study are shown in Table 1. Pcc strains were cultured at 28 °C on 1.4% nutrient agar (NA) or with shaking on modified Luria-Bertani (LB) medium, which contained half the recommended quantity of NaCl (5 g rather than 10 g of NaCl per litre). The IFO-802 medium was supplemented with 1% polypeptin, 0.2% yeast extract, 0.1% MgSO_4_ (pH 7.0), and 1.5% agar. Pcc isolates were identified to grow on the modified Drigalski agar medium [3]. Pcc H-rif-8-6 and TH22–10 were used as bacteriocin producers, while Pcc Ea1068 and SP33 were used as bacteriocin indicators. *E. coli* DH5α was used as a cloning host and was grown at 37 °C. *E. coli* BL21(DE3) was used as a protein expression host and was grown at 28 °C. *E. coli* strains were grown on normal LB medium with shaking. Rifampicin (50 mg/L), kanamycin (50 mg/L), ampicillin (50 mg/L), chloramphenicol (68 mg/L), and tetracycline (12.5 mg/L) were added to NA and LB agars whenever necessary. All bacterial growth densities were monitored by using a spectrophotometer at 595 nm (OD_595_).

**Table 1:** Bacteria and plasmids used in the study

Strain or plasmid	Description	Source
***Escherichia coli***		
DH5α	supE44ΔlacU169(Φ80lacZΔM15) hsdR17recA1 gyrA96thi-1relA1	[20]
BL21(DE3)	hsdS gal(λ*c*I*ts*857 *ind*1 Sam*7 nin5* l*ac*UV5-T7 gene 1)	Novagen
***Pectobacterium carotovorum*** **subsp.** ***carotovorum***		
H-rif-8-6	Pcc, Rif^r^, wild-type	Laboratory stock
TH22–10	H-rif-8-6, *CaroK1*::Tn*5*, Rif^r^, Kan^r^	This study
Ea1068	Pcc, wild-type	Laboratory stock
SP33	Pcc, wild-type	Laboratory stock
**Plasmid**		
pGEM T-Easy	Amp^r^; lacZ cloning vector	Promega
pEN3KI	*caroS3K* subcloned into PET32a	This study
pET32a	Amp^r^; expression vector with the N-terminal His-tag	Novagen
pES3KI	Derived from pEN3K; deleted series of Tag element in front of the expressed c*aroS3K*	This study
pES3I	Derived from pECS3I, the (His)_6_-Tag element was deleted	This study

*Kan*^*r*^ Kanamycin, *Cml*^*r*^ Chloramphenicol, *Rif*^*r*^ Rifampicin, *Amp*^*r*^ Ampicillin

### Preparation of genomic DNA, plasmid DNA and mRNA

The procedures of plasmid preparation, genomic DNA isolation, and DNA manipulation were performed as described by Sambrook et al. [21]. Exponentially growing cells of *E. coli* DH5α (OD_595_ of about 6.0) were harvested for RNA preparation. Total RNA was isolated using Trizol reagent (Invitrogen, USA) according to the manufacturer’s instructions. RNA was resuspended in diethylpyrocarbonate (DEPC)-treated water. The concentration of RNA was determined by OD_260_ absorption, and RNA was analysed by electrophoresis on 1.5% formaldehyde-morpholinepropanesulfonic-agarose gel.

### Antiserum preparation for Carocin S1K and Carocin S1I

The proteins Carocin S1K and Carocin S1I were expressed using the BL21/pAYL4 strain by overnight culture in LB medium. After centrifugation, the proteins contained in the supernatants were precipitated with 50% ethanol and loaded on preparative SDS-10% polyacrylamide gels. The bands corresponding to Carocin S1K and Carocin S1I were directly cut out, and the proteins were electroeluted for isolation. The protein was injected into a rabbit for antibody production.

### Western blotting

The western blot analysis was performed after electrophoretic transfer of proteins from SDS-PAGE gel to a poly(vinylidene difluoride) (PVDF) membrane. After electrophoresis, the proteins were electroblotted onto nitrocellulose in a semi-dry apparatus at 2 mA/cm^2^ for 20 min using a transfer buffer containing 40 mM glycine, 50 mM Tris, 0.4% SDS, and 10% methanol. The nitrocellulose was then saturated with gelatin, incubated with antibodies, and the blots were visualised using 3,3-diaminobenzidine (Sigma). Anti-CaroS1K or Anti-CaroS1I antibodies were diluted at 1:2500.

### Nest-PCR and reverse transcription-PCR

In this study, oligonucleotide DNA primers synthesised by MD Bio Inc. (Taipei, Taiwan), and Go-Taq DNA polymerase manufactured by Promega, USA were used for PCR amplification. The procedures described by Sambrook et al. [21] were followed for thermal asymmetric interlaced PCR (TAIL-PCR). On the other hand, the Reverse Transcription-PCR (RT-PCR) using AMV Reverse Transcriptase (Promega, USA) was carried out based on the instructions provided by the manufacturer.

One microgram (1 μg) of RNA was subjected to RT-PCR. CaroS3_re_1 was used as a reverse primer in first-strand cDNA synthesis. The RT mixtures were diluted and used as templates in a PCR reaction with the two primers, CaroS3_re_1 and CaroS3_for_1 (Table 2).

**Table 2:** Primers used in the study

Name	Sequence (5′ → 3′)
CaroS1_for2	5′-CGACTTGGATCCATGTCTGTAAAC
CaroS1_rev2	5′-GGCAGGAAGCTTACAGGGATATTA
CaroS3K_for_1	5′-GAGCTCCGTCGACAAGC
CaroS3K_re_1	5′-ATCTTGTTCCTGAAGC
CarocinS3KI_for	5′-GGATCCATGATTAAATACCG
CarocinS3kI_rev	5′-GAGCTCTTAGAGACCGTAT
5IHTGT2KI_forS	5′-ATGTTAAGTACCGTTTATA
5IHT32a2KI_forT	5′-GAAGGAGATATACATATGATTAAGTACCGTTTATA
5IHT32a3KI_revT	5′-ATGTATATCTCCTTCTTAAAGTTAAACAAAATTATTTC
5IHT32a4KI_revS	5′-TTAAAGTTAAACAAAATTATTTC
X4I_forS	5′-ATGATTAATTTTAAGGCAATG
X4I_forT	5′-GAAGGAGATATACATATGATTAATTTTAAGGCAATG

### Restriction DNA library screening and southern blots

The Southern blots were performed following the procedures described in the DIG Application Manual (Roche, USA). Using X4I_forS and X4I_forT (Table 2) as primers, the 390-bp DNA fragment (CAROS probe) from H-rif-8-6 was amplified. It was then subcloned into the pGEM-T Easy vector (Promega Inc., USA). Thereafter, it was labeled utilising a Random Primed DNA Labeling Kit (Roche Diagnostics, USA).

Digestion of the genomic DNA of the wild-type strain, H-rif-8-6, was done using different restriction endonucleases. Restriction sites were located outside the putative open reading frame. The samples were then subjected to electrophoresis and analysed with Southern blotting. After detection using the CAROS probe, the DNA from positive gel slices was purified. Subsequently, the DNA was cloned into pBR322 to provide the Carocin-producing plasmid pBRS3KI, which was isolated and detected with the CAROS probe.

### Bacteriocin assays

Bacteriocin production was examined employing the double-layer agar method of Fredericq [22]. The agar consists of hard (1.4%) and soft (0.65%) layers of IFO-802 agar medium. Growth inhibition zones around the colonies indicate the production of bacteriocin. Moreover, trypsin treatment was used for bacteriocin identification.

### Computer analysis of sequence data

The DNA nucleotide sequence and the deduced amino acid sequence of Carocin S1, Colicin E1, Colicin E2, and Colicin E3 were compared by the BLAST and FASTA programs of the National Center for Biotechnology Information server. Sequence data were compared by DNASIS MAX 3.0 software (Hitachi, Tokyo, Japan).

### Sub-cloning and transformation

About 4500-bp *BamH*I*-Hind*III digested DNA fragment, including the *caroS3K* and *caroS3I* genes, was amplified from pBRS3KI with primers of CarocinS3KI_for and CarocinS3KI_rev (Table 2) and a 2906-bp DNA fragment was subcloned into pET32a to give the plasmid pEN3KI. The pES3KI was obtained by excision of the Tag element between the rbs (ribosome binding site) and start code (for *caroS2K*) in pEN3KI using the SLIM method as previously described [13, 23, 24]. The 5IHT32a2KI_forT, 5IHTGT2KI_forS, 5IHT32a3KI_revT, and 5IHT32a4KI_revS primers were used. A 2636-bp fragment of the *caroS3K* gene was knocked-out by PCR to form the plasmid pES3I. The X4I_forS, X4I_forT, 5IHT32a3KI_revT, and 5IHT32a4KI_revS primers were used. Subsequently, pES3KI and pES3I were introduced into *E. coli* BL21 (DE3) cells.

Plasmids were introduced into Pcc strains using electroporation (1.25 kV/cm, 200 Ω, 25 μF) [21]. For heat-shock transformation, the competent cells of *E. coli* were prepared according to the method of Hanahan [20].

### Bacteriocin expression and bacteriocin activity assay

Bacterial strains (BL21/pET32a, BL21/pES3kI, and BL21/pES3I) in BSM medium were incubated in a sterilised stainless-steel box with stainless steel cover at 28 °C for 24 h under a dark condition. After centrifugation, the medium without cells was removed.

The nucleotidase activity was confirmed by adding 500 ng/1 μl genome DNA solution from strain Ea1068 into 10 μl of the protein solution and incubated at 28 °C for 90 min. An equal quantity of genomic DNA was digested with *EcoR*I at 28 °C for 90 min. Samples were then subjected to electrophoresis on 1% agarose gel.

The bacteriocin activity was determined by adding 10 μl of the protein solution to an indicator plate containing Ea1068 strain growing on soft IFO-802 medium containing 0.65% agar at 28 °C for 1 day. Growth inhibition zones at the point of addition were considered an indication of Carocin S3 activity.

The ribonuclease activity was determined following an assay procedure provided by the manufacturer (Promega, USA). Firstly, total DNA was treated with calf intestinal alkaline phosphatase at 55 °C for 30 min. The reaction was then arrested by adding 5 mM nitrilotriacetic acid. Equal volumes of phenol and chloroform were used to extract the RNA. An aliquot of phosphatase-treated RNA was 5′-^32^P-labeled at 37 °C for 30 min. The labeling was done by incubating the RNA with a mixture of [γ-32P] ATP, T4 polynucleotide kinase (Promega Inc., USA), and reaction buffer in nuclease-free water [24]. The 3′-labeling of RNA then followed using [5′-^32^P] Cytidine 3′,5′-bisphosphate (pCp) and T4 RNA ligase (Promega, USA) [25]. MicroSpin G-25 columns (GE Healthcare, USA) were used to purify the mixture. Subsequently, aliquots of the purified labelled-RNA were incubated with and without Carocin S3 at 28 °C for 60 min.

To measure its activity, Carocin S3I was pre-mixed with an equal amount of Carocin S3K. The mixtures were subjected to electrophoresis on a 9% polyacrylamide gel (19:1) containing 7 M urea, 50 mM Tris, 50 mM boric acid, and 1 mM EDTA, pH 8.3. All samples were electrophoresed at 15 °C by PROTEIN II xi (BioRad, USA).

### Protein purification

For rough protein isolation, bacterial strains (BL21/pET32a, BL21/pES3kI and BL21/pES3I) in BSM were incubated in the dark using a sterilised stainless-steel box with steel cover at 28 °C for 24 h. After centrifugation, the medium without cells was removed. Ammonium sulfate was added to 80% saturation to precipitate the protein, and the precipitate was collected on a 0.45-μm cellulose filter. One milligram of precipitated protein was dissolved in 100 μl of bacteriocin buffer (0.1 M Tris [pH 7.5], 0.01 M dithiothreitol, and 0.5 M MgCl_2_).

For bacteriocin isolation, the transformant cells of BL21, harbouring pES3KI or pES3I, were grown in 500 ml to an OD_595_ of 0.5 ~ 0.6. The cells were induced with isopropyl-β-D-thiogalactopyranoside (IPTG; final concentration, 0.1 mM; at 25 °C for 14 h). Subsequently, the cells were pelletised in a loading buffer (20 mM Tris-HCl, 28 mM NaCl, PH 8.0), and the pellets were sonicated (10 cycles of 9 s with 9-s intervals). The cell extract was collected and applied to a Q-Sepharose column (Merck, USA). The fraction was eluted by a 5% ~ 15% elution buffer (20 mM Tris-HCl, 1.4 M NaCl, PH 8.0).

BL21/pES3KI was precipitated with 40–50% ammonium sulfate, and was resuspended in buffer A, which contains 30 mM NaCl and 20 mM Tris-Cl, pH 8.0. On the other hand, BL21/pES3I was precipitated with 70% ammonium sulfate and likewise resuspended in buffer A. All protein concentrations were determined by the Bradford assay (Amresco, USA).

### Antibiotic activity of Carocin S3

Ea1068 were grown overnight in LB medium at 28 °C. The overnight cultures were diluted to a density of approximately 10^5^ CFU/ml. The activity of increasing concentrations of Carocin S3 was assessed by incubating it with the cells in suspension at 28 °C for 60 min. Equal molar ratio of Carocin S3I and Carocin S3K were pre-mixed. The reaction mixtures were then spread onto LB agar plates and incubated at 28 °C for 16 h. Finally, the colonies were counted to determine the antibiotic activity of Carocin S3.

### Electrospray ionization mass spectrometry for Carocin S3 and Carocin S3I molecular weight assay

ESI-MS spectra were obtained on a Fisons VG platform using 37.5% methanol as the sample solvent for Carocin S3K and Carocin S3I. Samples were dialysed against water to remove salts and mixed with the sample solvent before analysis. Approximately 1 nmol of protein was analysed per run. Data were acquired over the m/z range 800 to 2000, with ten scans averaged and processed using the supplier’s MassLynx software. The standard errors quoted in the text represent the errors from three separate mass determinations for each sample.

## Supplementary information

**Additional file 1 Supplementary Fig. 1.** Alignment of Carocin S3K with homologous bacteriocins.

## Data Availability

The datasets used and analysed during the current study are available from the corresponding authors on reasonable request.

## Abbreviations

HMWB: High-Molecular-Weight Bacteriocins
LMWB: Low-Molecular-Weight Bacteriocins
Pcc: *Pectobacterium carotovorum* subsp. c*arotovorum*
TAIL-PCR: Thermal Asymmetric Interlaced Polymerase Chain Reaction
