# Supplementary material for: Identification and characterization of the bacteriocin Carocin S3 from the multiple bacteriocin producing strain of Pectobacterium carotovorum subsp. carotovorum
Source: BMC Microbiol. 2020 Sep 1;20:273. doi: 10.1186/s12866-020-01955-9 (PMC7461348; doi:10.1186/s12866-020-01955-9)
Supplement: Supplementary file 1 — Additional file 1 Supplementary Fig. 1. Alignment of Carocin S3K with homologous bacteriocins. [file 12866_2020_1955_MOESM1_ESM.pdf]

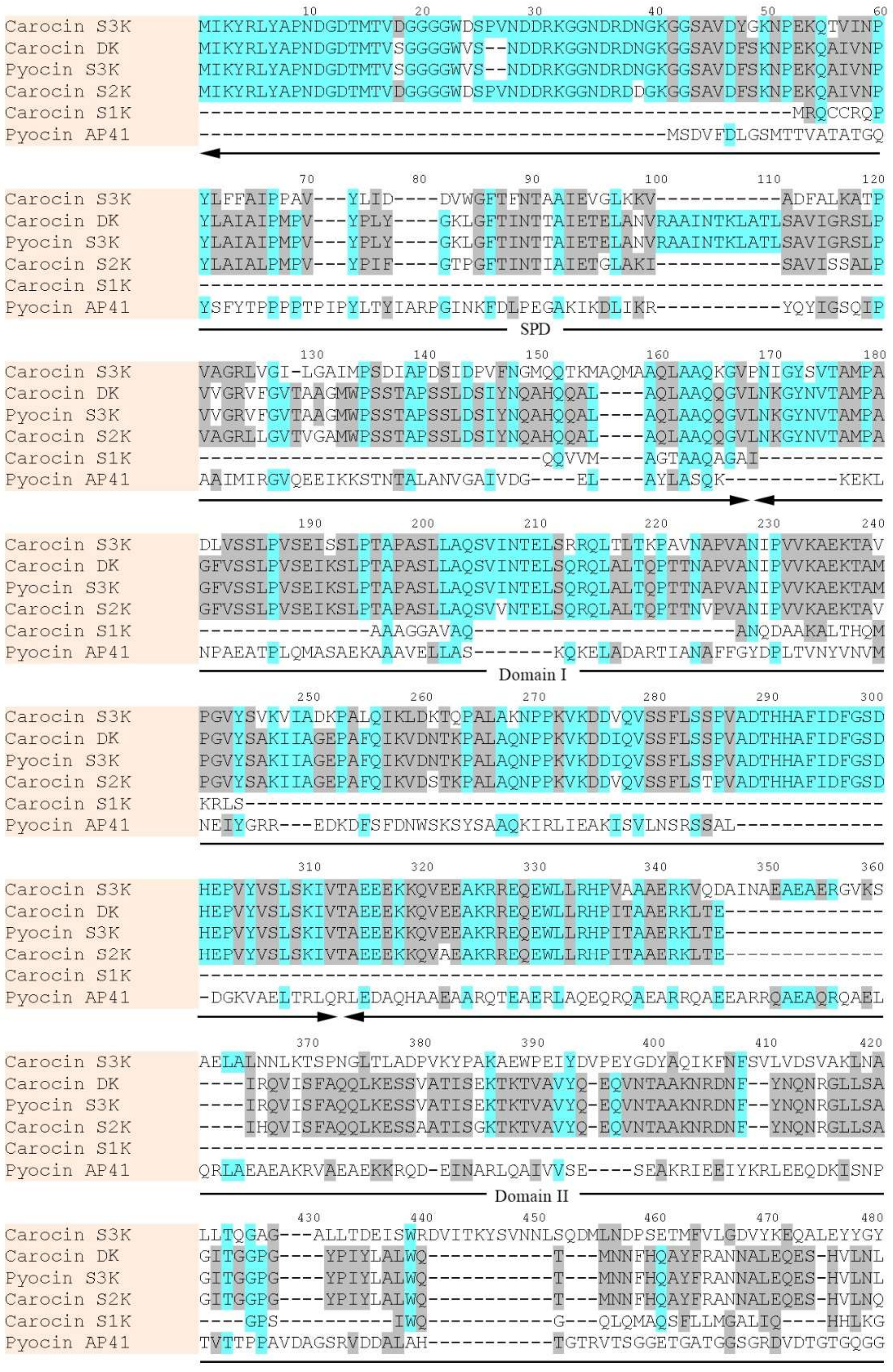

Carocin S3K
Carocin DK
Pyocin S3K
Carocin S2K
Carocin S1K
Pyocin AP41
SPD
Domain I
Domain II

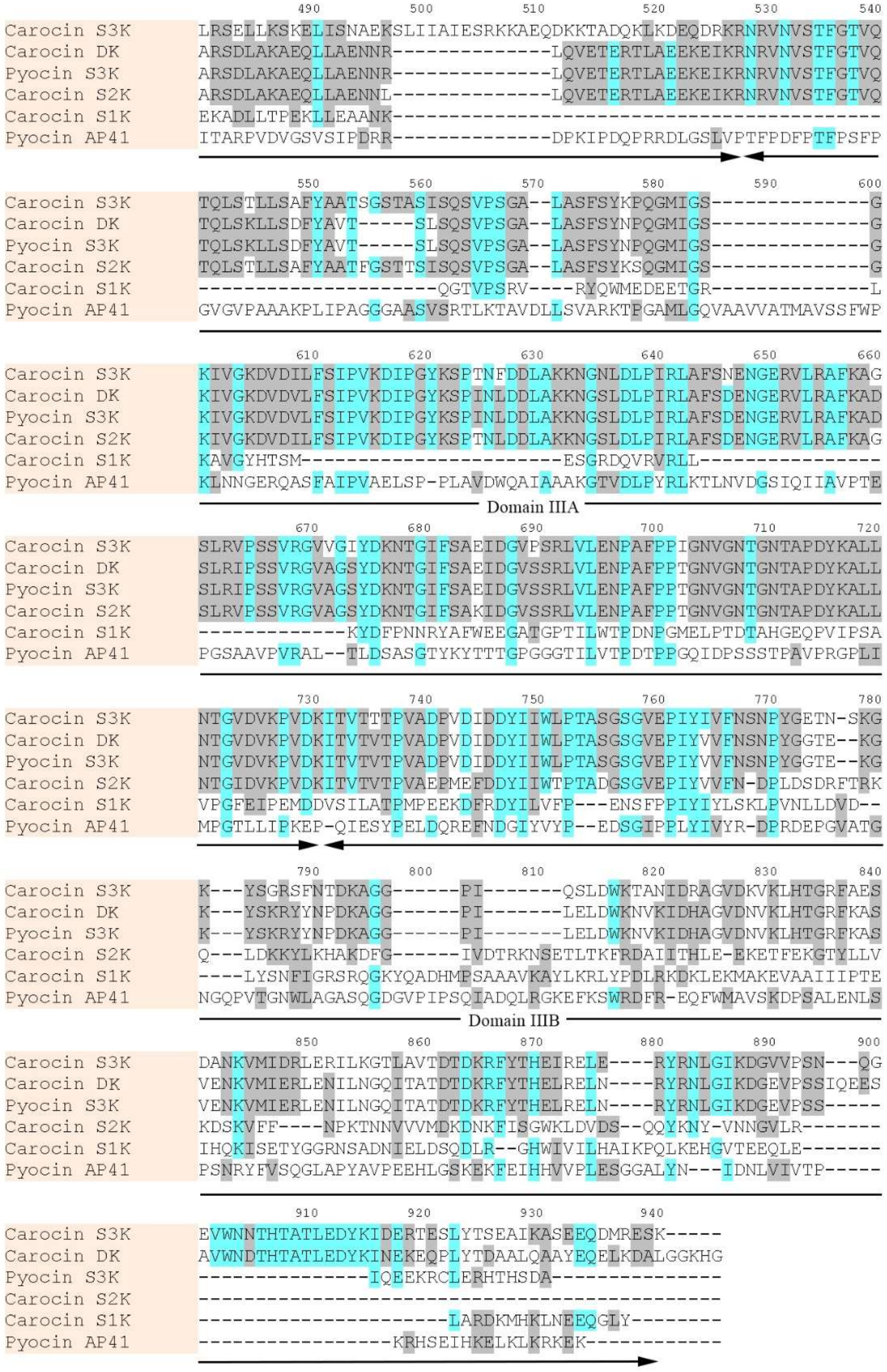
490 500 510 520 530 540
Carocin S3K LRSELLKSKELISNAEKSLIIAIESRKKAEQDKKTADQKLKDEQDRKRNRVNVSTFGTVQ
Carocin DK ARSDLAKAEQLLAENNR--------------LQVETERTLAEEKEIKRNRVNVSTFGTVQ
Pyocin S3K ARSDLAKAEQLLAENNR--------------LQVETERTLAEEKEIKRNRVNVSTFGTVQ
Carocin S2K ARSDLAKAEQLLAENNL--------------LQVETERTLAEEKEIKRNRVNVSTFGTVQ
Carocin S1K EKADLLTPEKLLEAANK---
Pyocin AP41 ITARPVDVGSVSIPDRR--------------DPKIPDQPRRDLGSLVPTFPDFPTFPSFP
550 560 570 580 590 600
Carocin S3K TQLSTLLSAFYAATSGSTASISQSVPSGA--LASFSYKPQGMIGS--------------G
Carocin DK TQLSKLLSDFYAVT-----SLSQSVPSGA--LASFSYNPQGMIGS--------------G
Pyocin S3K TQLSKLLSDFYAVT-----SLSQSVPSGA--LASFSYNPQGMIGS--------------G
Carocin S2K TQLSTLLSAFYAATFGSTTSISQSVPSGA--LASFSYKSQGMIGS--------------G
Carocin S1K ---------------------QGTVPSRV----RYQWMEDEETGR--------------L
Pyocin AP41 GVGVPAAAKPLIPAGGGAASVSRTLKTAVDLLSVARKTPGAMLGQVAAVVATMAVSSFWP
610 620 630 640 650 660
Carocin S3K KIVGKDVDILFSIPVKDIPGYKSPTNFDDLAKKNGNLDLPIRLAFSNENGERVLRAFKAG
Carocin DK KIVGKDVDVLFSIPVKDIPGYKSPINLDDLAKKNGSLDLPIRLAFSDENGERVLRAFKAD
Pyocin S3K KIVGKDVDVLFSIPVKDIPGYKSPINLDDLAKKNGSLDLPIRLAFSDENGERVLRAFKAD
Carocin S2K KIVGKDVDILFSIPVKDIPGYKSPTNLDDLAKKNGSLDLPIRLAFSDENGERVLRAFKAG
Carocin S1K KAVGYHTSM-----------------------ESGRDQVRVRLL----------------
Pyocin AP41 KLNNGERQASFAIPVAELSP-PLAVDWQAIAAAKGTVDLPYRLKTLNVDGSIQIIAVPTE
Domain IIIA
670 680 690 700 710 720
Carocin S3K SLRVPSSVRGVVGIYDKNTGIFSAEIDGVPSRLVLENPAFPPIGNVGNTGNTAPDYKALL
Carocin DK SLRIPSSVRGVAGSYDKNTGIFSAEIDGVSSRLVLENPAFPPTGNVGNTGNTAPDYKALL
Pyocin S3K SLRIPSSVRGVAGSYDKNTGIFSAEIDGVSSRLVLENPAFPPTGNVGNTGNTAPDYKALL
Carocin S2K SLRVPSSVRGVAGSYDKNTGIFSAKIDGVSSRLVLENPAFPPTGNVGNTGNTAPDYKALL
Carocin S1K -------------KYDFPNNRYAFWEEGATGPTILWTPDNPGMELPTDTAHGEQPVIPSA
Pyocin AP41 PGSAAVPVRAL--TLDSASGTYKYTTTGPGGGTILVTPDTPPGQIDPSSSTPAVPRGPLI
730 740 750 760 770 780
Carocin S3K NTGVDVKPVDKITVTTTPVADPVDIDDYIIWLPTASGSGVEPIYIVFNSNPYGETN-SKG
Carocin DK NTGVDVKPVDKITVTVTPVADPVDIDDYIIWLPTASGSGVEPIYVVFNSNPYGGTE--KG
Pyocin S3K NTGVDVKPVDKITVTVTPVADPVDIDDYIIWLPTASGSGVEPIYVVFNSNPYGGTE--KG
Carocin S2K NTGIDVKPVDKITVTVTPVAEPMEFDDYIIWTPTADGSGVEPIYVVFN-DPLDSDRFTRK
Carocin S1K VPGFEIPEMDDVSILATPMPEEKDFRDYILVFP---ENSFPPIYIYLSKLPVNLLDVD--
Pyocin AP41 MPGTLLIPKEP-QIESYPELDQREFNDGIYVYP--EDSGIPPLYIVYR-DPRDEPGVATG
790 800 810 820 830 840
Carocin S3K K---YSGRSFNTDKAGG------PI-------QSLDWKTANIDRAGVDKVKLHTGRFAES
Carocin DK K---YSKRYYNPDKAGG------PI-------LELDWKNVKIDHAGVDNVKLHTGRFKAS
Pyocin S3K K---YSKRYYNPDKAGG------PI-------LELDWKNVKIDHAGVDNVKLHTGRFKAS
Carocin S2K Q---LDKKYLKHAKDFG------IVDTRKNSETLTKFRDAIITHLE-EKETFEKGTYLLV
Carocin S1K ----LYSNFIGRSRQGKYQADHMPSAAAVKAYLKRLYPDLRKDKLEKMAKEVAAIIIPTE
Pyocin AP41 NGQPVTGNWLAGASQGDGVPIPSQIADQLRGKEFKSWRDFR-EQFWMAVSKDPSALENLS
Domain IIIB
850 860 870 880 890 900
Carocin S3K DANKVMIDRLERILKGTLAVTDTDKRFYTHEIRELE----RYRNLGIKDGVVPSN---QG
Carocin DK VENKVMIERLENILNGQITATDTDKRFYTHELRELN----RYRNLGIKDGEVPSSIQEES
Pyocin S3K VENKVMIERLENILNGQITATDTDKRFYTHELRELN----RYRNLGIKDGEVPSS-----
Carocin S2K KDSKVFF----NPKTNNVVVMDKDNKFISGWKLDVDS--QQYKNY-VNNGVLR-------
Carocin S1K IHQKISETYGGRNSADNIELDSQDLR--GHWIVILHAIKPQLKEHGVTEEQLE-------
Pyocin AP41 PSNRYFVSQGLAPYAVPEEHLGSKEKFEIHHVVPLESGGALYN---IDNLVIVTP-----
910 920 930 940
Carocin S3K EVWNNTHTATLEDYKIDERTESLYTSEAIKASEEQDMRESK-----
Carocin DK AVWNDTHTATLEDYKINEKEQPLYTDAALQAAYEQELKDALGGKHG
Pyocin S3K ---------------IQEEKRCLERHTHSDA---------------
Carocin S2K --
Carocin S1K ----------------------LARDKMHKLNEEQGLY--------
Pyocin AP41 -----------------KRHSEIHKELKLKRKEK------------
